# Potential of *Garra rufa* as a novel high-temperature resistant model fish: a review on current and future approaches

**DOI:** 10.1186/s40851-025-00249-0

**Published:** 2025-02-27

**Authors:** Yasuhito Shimada, Baki Aydın, Koto Kon-Nanjo, Kiki Syaputri Handayani, Victor David Nico Gultom, Oleg Simakov, Fahrurrozi, Tetsuo Kon

**Affiliations:** 1 Mie University Zebrafish Research Center, 2-174 Edobashi, Tsu, Mie 5148572 Japan; 2 Department of Integrative Pharmacology, Mie University Graduate School of Medicine, 2-174 Edobashi, Tsu, Mie 5148572 Japan; 3 Department of Aquaculture, Faculty of Fisheries, Akdeniz University, Antalya, 07070 Türkiye; 4 Department of Neurosciences and Developmental Biology, University of Vienna, 1030 Vienna, Austria; 5 Research Center for Marine and Land Bioindustry, Research Organization for Earth Sciences and Maritime, National Research and Innovation Agency, Teluk Kodek, Pemenang, West Nusa Tenggara 83352 Indonesia; 6 Department of Integrative Pharmacology, Mie University Graduate School of Medicine, 2-174 Edobashi, Tsu, Mie 514-8507 Japan

**Keywords:** High-temperature tolerance, Red garra, Cyprinid, Ichthyotherapy, Small fish model, Genome assembly

## Abstract

*Garra rufa*, commonly known as the “doctor fish”, is a freshwater cyprinid native to warm regions of the Middle East. Since the late twentieth century, it has been widely utilized in spas for alternative therapeutics and fish pedicures (or manicures) for dermatological diseases such as psoriasis and eczema. Owing to its unique characteristics, there is growing interest in exploring various applications of *G. rufa*. This review provides a comprehensive summary of the phylogenetic position, ecology, biological characteristics, and breeding methods of *G. rufa*, and provides insights into its use as a therapeutic fish. Notably, the ability of *G. rufa* to thrive in high-temperature environments exceeding 37 °C distinguishes it from other cyprinids and suggests its potential as a model for human diseases, such as human infectious diseases, and in use in cancer xenograft models for high-throughput drug screening. The ongoing genome sequencing project for *G. rufa* aims to elucidate the mechanisms underlying its high-temperature tolerance and offers valuable genomic resources. These efforts have resulted in significant advances in fish aquaculture, species conservation, and biomedical research.

## Introduction

*Garra rufa* (Heckel, 1843), commonly known as the “doctor fish” or red garra, is a small freshwater teleost fish (usually less than 15 cm in length and 40 g in weight) that is native to rivers, streams, and lakes in the Middle Eastern regions, including Türkiye, Syria, Iraq, and Iran. It belongs to the family Cyprinidae, which is a large family with more than 150 species, including other carp-related species, such as zebrafish and goldfish [1]. Over the past two decades, over 40 new species have been discovered in *Garra*. As of 31 March 2024, the academic database Web of Science (https://www.webofscience.com/) listed 302 articles under the topic heading “*Garra*.” Of these, 223 articles specifically reported on *Garra* fish, while the remaining papers focused on subjects such as the "*Garra rufa* optimization-assisted deep learning model,” or contained minimal references to *Garra* fish in their content. Of these 223 articles, 56 articles (approximately 25%) focused on *G. rufa*, highlighting its significance as the most-studied species within the genus. The second most researched species were *G. lamta* and *G. barreimiae*, each with nine papers (approximately 4%).

The biological characteristics of *G. rufa* are fascinating and contribute to its unique role in biotherapy in natural ecosystems and human therapeutic practices. The fish are known for their unique behavior of feeding on dead keratinized cells of human skin, an activity popularly referred to as fish pedicure or ichthyotherapy, which has made these fish popular in the tourism and health industries as natural solutions for acne, psoriasis, and eczema [2–4].

Their resilience in degraded river environments, tolerance to high temperatures, which enables them to thrive at spa water temperatures, and omnivorous diet, which includes algae, detritus, small animals, and dead human skin, are believed to facilitate habitat expansion. Originating in the Middle East, *G. rufa* has been transported to various regions for ichthyotherapy to treat skin diseases, leading to some populations becoming invasive [5].

This review summarizes recent insights from research on *G. rufa*, covering its geographical distribution, habitat, culture, diseases, and therapeutic applications. In addition, we highlight the potential of *G. rufa* as a novel animal model in human disease research, extending beyond its traditional role in ichthyotherapy. This potential is largely attributed to its high-temperature resistance, suggesting that *G. rufa* could serve as a novel small-fish model alongside zebrafish and medaka.

## Phylogenetic relationship between *G. rufa* and other small teleost fish

**Fig. 1:**
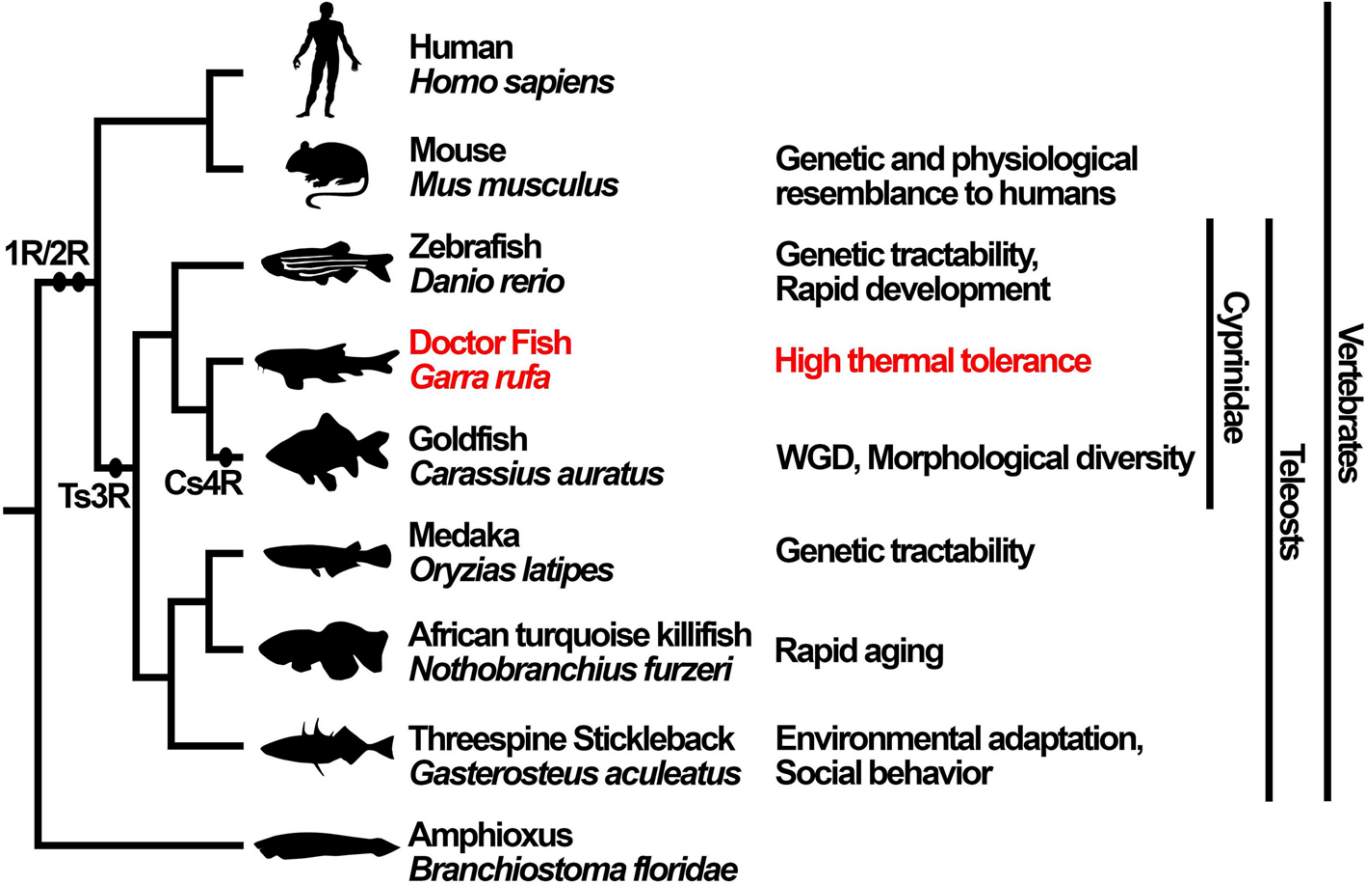
Phylogenetic relationship between humans, *G. rufa*, and other model organisms. The phylogenetic relationships between humans, *G. rufa*, and other common vertebrate model animals, and amphioxus are illustrated. The common and scientific names of each animal are displayed to the right of each animal silhouette, and further to the right, a brief description of the characteristics of each animal as a model organism is provided. The topology of the cladogram is based on previous studies [22, 23]. WGD, whole-genome duplication; 1R/2R, first and second rounds of WGD; Ts3R, teleost-specific third-round WGD; Cs4R, common carp and goldfish-specific fourth round WGD [17–19]. Animal silhouettes are from PhyloPic (www.phylopic.org)

Teleosts are a large group of Actinopterygii (ray-finned fish) characterized by a specific genome structure resulting from teleost-specific third-round whole-genome duplication (Ts3R), which could contribute to their evolutionary success[6–9] (Fig. 1). Small teleost fish are used widely in biomedical research because of their high experimental throughput, their closer genetic proximity to humans than invertebrate model organisms such as *Drosophila* and nematodes, and the relatively lower ethical barriers to their experimental use compared to those for mammalian models, such as mice [10–12]. Each small teleost fish model has its own characteristics; zebrafish (*Danio rerio*) and medaka (*Oryzias latipes*) are relatively easy to manipulate genetically [13–16], goldfish (*Carassius auratus*) has undergone a recent whole-genome duplication (Cs4R) [17–19], the threespine stickleback (*Gasterosteus aculeatus*) allows for the analysis of complex social behavior [20], and the African turquoise killifish (*Nothobranchius furzeri*) serves as a unique model for studying aging [21].

*G. rufa* belongs to the Cyprinidae family and is similar to zebrafish and goldfish. The taxonomy of the subfamilies within the family Cyprinidae is debated, with the number reported of subfamilies varying depending on the study. Subfamilies such as Danioninae, Cyprininae, Labeoninae, and Barbinae have been defined; however, consensus has not yet been reached on their phylogenetic relationships [22, 24–26]. A recent mitochondrial genome analysis showed that *G. rufa* belongs to the Labeoninae subfamily within the family Cyprinidae, along with the mud carp (*Cirrhinus molitorella*) and rohu (*Labeo rohita*) [23]. In addition to *G. rufa*, the genus *Garra* includes several additional species, such as *G. jordanica*, *G. ghorensis*, and *G. nana* [27, 28]. Through mitochondrial DNA analyses, they have been shown to be genetically differentiated based on their geographical distribution [27–29].

## Traits of *G. rufa*

### Appearance

**Fig. 2:**
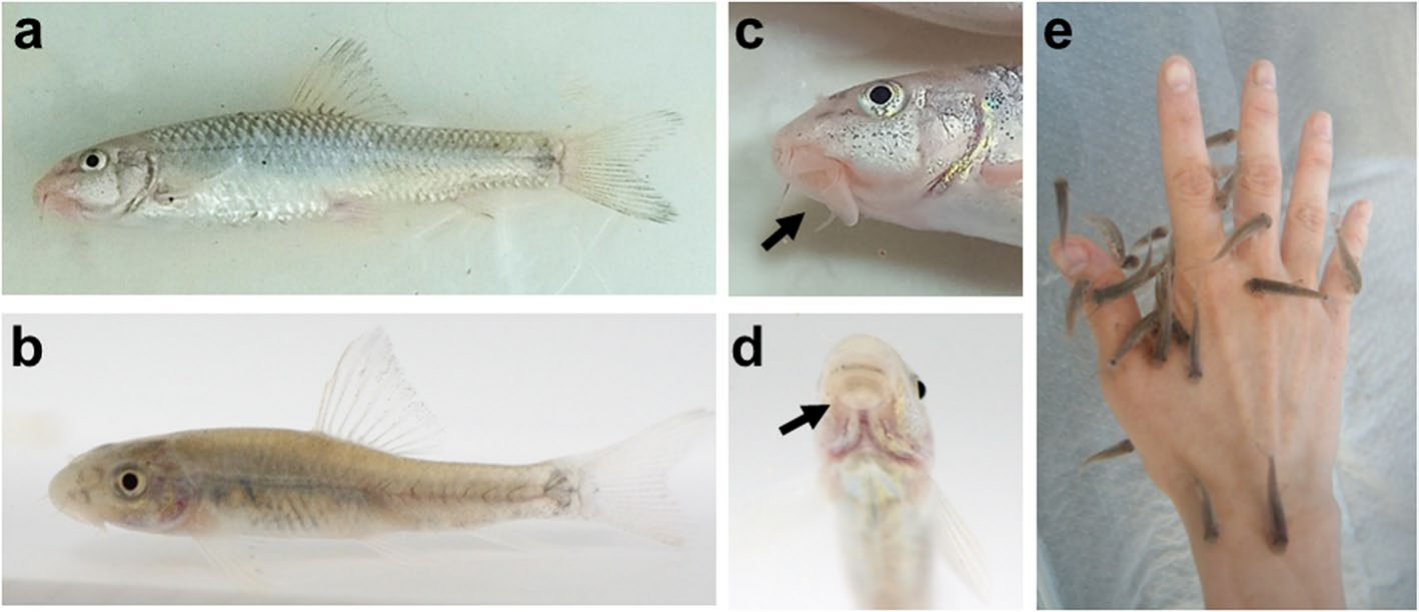
Gross appearance of *G. rufa*. **a**-**b** Gross appearance of an adult (**a**) and young (**b**) *G. rufa*. (**c**) Lateral view of mouth of adult *G. rufa*. (**d**) Frontal view of young mouth. The mouth (black arrows) in *G. rufa* has the additional function to adhere to bottom surfaces and to scoop up food particles. (**e**) *G. rufa* engaging in ichthyotherapy

*G. rufa* is relatively small, measuring approximately 15 cm in length when fully grown [30]. They reach sexual maturity at the age of one year [31]. In natural habitats, spawning occurs from May to August. They generally exhibit a grayish-brown hue, which helps them to blend into their natural riverbed environments (Fig. 2a and b). Their bodies may display subtle patterns or markings, and may have a paler underbelly. *G. rufa* has a broad head and an elongated body that tapers towards its tail. They have rounded fins, including a dorsal fin set midway along the back and a rounded tail fin (Fig. 2a and b). Their scales are small and tightly fitted, giving them a smooth texture; the scales take seven distinct shapes, and otoliths take three different forms [32]. An adhesive disk or organ near the mouth is positioned on the lower part of the fish (Fig. 2c and d). Fish can readily locate and eat their feed using their ventrally positioned mouth because of the sticky disc located on the bottom lip, which is used to attach to habitat surfaces, including rocks and stones [33]. This anatomical feature allows *G. rufa* to adhere to human skin and feed on dead keratinocytes (Fig. 2e).

### Habitat

*G. rufa* is found in many freshwater streams in the Asi, Tigris-Euphrates, Ceyhan, and Seyhan rivers in Türkiye, as well as in freshwater bodies in Syria, Iraq, Iran, and Jordan [34, 35]. Fish thrive in these river basins because of the suitable climatic and water conditions, including warm and slightly mineralized waters. *G. rufa* is a benthic fish that prefers shallow, fast-moving, warm waters with rocky or sandy bottoms conducive to its feeding habits. The native environments of different *Garra* species differ in several ways. For example, *G. rufa* is dominant in turbid, large, and relatively deep streams, whereas *G. rezai* is dominant in clear, shallow, and small streams. In the Tigris–Euphrates River, which is the natural habitat of *G. rufa*, other *Garra* species are present, such as *G. rezai* and *G. variabilis*, which are very similar in external anatomy and are genetically closely related [36]. Therefore, in addition to phenotypic characteristics, analyses using genomic and mitochondrial DNA are important for identifying *Garra* species.

### Breeding

The population of *G. rufa* in their natural habitats is insufficient to meet the demand for ichthyotherapy and other purposes, putting pressure on their natural stocks [37]. Habitat degradation caused by climate change, anthropogenic activities, and increased pollution levels in aquatic environments also affects *G. rufa* populations [38, 39]. Therefore, the cultivation of *G. rufa* is important for protecting populations and creating a future for sustainable environments.

Water temperature is a critical factor in aquaculture. Generally, *G. rufa* is resistant to both low (3–7 °C) and high (38–43 °C) temperatures, and thus their thermal tolerance polygon values are high [40]; the Kangal Fish Spring in Türkiye, one of the natural habitats of this fish, has average water temperatures of 35 °C [4]. This is a significant advantage for cultivating *G. rufa* in tropical regions. However, to maintain *G. rufa* cultivation, sudden and frequent temperature changes should be avoided [40]. Sudden water temperature changes in aquaculture can result in water quality deterioration, hypoxia, nutritional abnormalities, parasitic infections, and bacterial or fungal pathogens.

*G. rufa* can be reared in environments of varying sizes. On the larger end, *G. rufa* can be kept in large tanks with a depth of several tens of centimeters and dimensions from 1 to 2 m, which mimic their natural habitat. However, based on our experience, it is also feasible to maintain them in small-scale laboratory settings. For instance, we house 5–10 young *G. rufa* (~ 6 mpf) individuals in standard 2 L zebrafish tanks with no signs of aggressive or abnormal behavior. As *G. rufa* matures, reaching an adult body length of approximately 10 cm, it is preferable to rear them in standard 360 L tanks (100 cm × 60 cm × 60 cm). As *G. rufa* is a bottom-dwelling fish in natural environments, tanks with large bottom areas are considered ideal.

To date, few studies have been conducted on *G. rufa* culture, and the optimal conditions for its cultivation are gradually being defined [31, 41]. The fertilized eggs of *G. rufa* are non-adhesive, sink to the bottom of the tank, and must be promptly relocated to prevent the parent fish from eating them. The eggs can be sterilized prior to incubation by immersion in 5 mg/L methylene blue for 40–60 min. The spawning frequency of *G. rufa* is once every 15–30 days, with 57–314 eggs produced with an average diameter of 2.83 mm. When the fish were spawned in aquariums at water temperatures of 28 °C, 85–90% of the eggs hatched in 35 h, larvae opened their mouths in three days and started feeding exogenously, and began swimming by the fourth day post-hatching. Additionally, the larval stage of this species lasts approximately 15 days in water with temperature ranges between 24 °C and 32 °C.

Although phytoplankton are the primary food source of *G. rufa*, these fish also receive nutrients from bacteria and zooplankton in their native habitat [42]. Compared with zebrafish, *G. rufa* consumes a higher amount of feed (3.2% of their body weight per day) [31]. This indicates that *G. rufa* has high metabolic activity, which may contribute to survival in high-temperature environments.

### Diseases

Infectious diseases are among the most significant yet manageable diseases affecting *G. rufa*. Among parasites, *Clinostomum complanatum* has been reported to infect *G. rufa* in freshwater in Türkiye [43], whereas *Paradiplozoon bingolensis* has been reported to infect *G. rufa* in the Göynük River in Türkiye [44]. Pathogenic bacteria such as *Aeromonas sobria* [45] cause significant damage to *G. rufa* during cultivation and trade. Pathogens introduced from the natural environment into aquaculture facilities can cause mortality, growth retardation, and decreased feed conversion rates in cultured fish, making proper quarantine essential. In *G. rufa* aquaculture, the use of sedative and anesthetic substances such as clove oil and 2-phenoxyethanol is recommended to reduce stress during transportation, parasite inspection, and other critical routine farming operations [46, 47].

## Conventional roles of *G. rufa* in human society

### G. rufa as “doctor fish”

*G. rufa*, which has gained international recognition as the “doctor fish,” makes a unique contribution to the treatment of skin disorders, particularly psoriasis. This natural exfoliation process, often referred to as “ichthyotherapy,” involves patients immersing affected areas of their skin in water basins containing *G. rufa* (Fig. 2e). The fish then feed on the dead skin, providing a therapeutic benefit. The use of the “doctor fish” as a complementary treatment for skin conditions is based on the ability of the fish to exfoliate, potentially improving the overall health of the skin. Patients with psoriasis, a chronic autoimmune skin condition characterized by red, flaky, and crusty patches of skin covered with silvery scales, have reported significant relief and reduction in symptoms following treatment with *G. rufa* [2, 48, 49]. However, it is important to note that, although many individuals have experienced positive outcomes from ichthyotherapy, this practice should be considered a complementary approach rather than a standalone treatment. The scientific community continues to study the extent of its benefits and the underlying mechanisms.

Although ichthyotherapy with *G. rufa* offers some benefits, it also poses some challenges. Concerns regarding the potential transmission of infections from these fish have escalated since 2000. Volpe et al. reported that multiple pathogens were present in both fish and humans [50, 51]. Notably, methicillin-resistant *Staphylococcus aureus* [52] and *Mycobacterium marinum* infections [53] following ichthyotherapy have been documented. Various types of bacteria (*Aeromonas* spp., *Vibrio* spp., *P. aeruginosa*, and *Mycobacterium* spp.) have been detected in fish spa ponds, which may have originated from water, fish, or humans [45]. During fish therapy, the water containing the fish can transmit infections from one person to another. However, the transmission of the infection through contaminated water to healthy individuals is limited to hand and foot fish spas [54]. The sanitary conditions of the water and the health status of the fish are critical for ensuring the safety and efficacy of treatment, including the use of certain water disinfectants [55].

Despite these concerns, the use of *G. rufa* in the treatment of skin diseases remains a topic of interest for dermatologists and patients seeking alternative or complementary therapies. As research advances, a clearer understanding of the benefits, limitations, and best practices associated with ichthyotherapy may emerge, potentially solidifying its application in the broader context of dermatological treatment.

### *G*. *rufa* as ornamental fish

Ornamental fish are bred and maintained primarily for their aesthetic appeal, often in aquariums or artificial ponds. Examples include goldfish (*Carassius auratus*), common carp (*Cyprinus carpio*), medaka (*Oryzias latipes*), betta fish (*Betta splendens*), and various tropical fish species. The annual turnover of ornamental fisheries, which generates up to $15 billion in revenue, is an important commercial activity supporting one of the most popular leisure activities worldwide [56]. Ornamental fish often exhibit unique phenotypes, such as diverse body colors and shapes. Studying the molecular mechanisms underlying these phenotypes in ornamental fish can provide insights into the fundamental biological principles of vertebrates, highlighting their value as experimental models [17, 19, 57–59].

The *Garra* species *G. lissorhynchus*, *G. spilota*, *G. culiciphaga*, and *G. rufa* are also known as ornamental or aquarium fish (www.aquariumglaser.de). Although *G. rufa* are bottom-dwelling fish, they are beneficial to breeders and are preferred by many aquarists because they help control algae and accumulation of excess feed in aquariums. *G. rufa* is imported and utilized in many countries. Many are cultured in Indonesia [54, 60], with active cultivation on the island of Java. Producers also trade this fish in marketplaces from East to West Java. Producers from West Java offer lower prices than those from East Java. Most producers are from Bekasi. Online vendors sell *G. rufa* in the size range of 1–3 cm at a price range 0.06 of 0.19 USD.

### *G*. *rufa* as human nutrition

*G. rufa* is also consumed by humans and is considered edible by the local people of Oman [61] and Türkiye [62, 63]. The flesh of *G. rufa* has been demonstrated to contain a high proportion of dietary fatty acids such as oleic acid (18:1ω9), eicosapentaenoic acid (20:5ω3), docosahexaenoic acid (22:6ω3), arachidonic acid (20:4n-6), omega 3 (n-3)/omega 6 (n-6), and total monounsaturated fatty acids [64]. Seasonal variation in water temperature and feed composition affect total lipid and fatty acid content [64]. These fatty acids promote human health by improving dyslipidemia, preventing thrombosis, and exerting neuroprotective effects. Therefore, *G. rufa* has potential not only as a simple source of calories, but also as a functional food. In addition, because of its ease of farming and high heat tolerance, *G. rufa* may become an important alternative food source for humans in the near future, as global warming progresses [65].

## Emerging roles of *G. rufa* in human health care and medical research

### Expansion of medical use as “doctor fish”

The medical and therapeutic applications of *G. rufa*, particularly in dermatology and alternative medicine, have broadened considerably. In addition to its well-documented use in the treatment of psoriasis, *G. rufa* is used to assist in the management of eczema and various dry skin conditions. The removal of dead skin layers potentially reduces discomfort and improves the efficacy of moisturizing treatments.

The potential of *G. rufa* in enhancing wound healing has garnered increasing interest. Although research is ongoing, it has been posited that these fish may aid in wound cleaning by eliminating dead skin, thereby lowering infection risk and accelerating the healing process. Notably, the saliva of *G. rufa* is believed to contain components that promote wound healing, including antimicrobial agents [3, 60]. Although these specific components have not yet been identified, similar beneficial substances, mainly antimicrobial peptides, have been detected in the skin and mucus of various fish species [66–68], suggesting the possibility of analogous compounds in *G. rufa*. This attribute may extend its medical applications beyond conventional dermatology. In addition, *G. rufa* treatment is associated with stress relief and benefits to mental well-being. The unique sensation of fish nibbling is often described as gentle tickling, which induces relaxation and enhances the overall spa experience. A clinical trial of ichthyotherapy for psoriasis at Kangal Hot Springs in Türkiye reported a reduction in patient stress and enhancement in psychological well-being [4]. This suggests that the therapy could be applicable not only to dermatological diseases but also to various stress-related illnesses and mental disorders.

### Novel fish with high-temperature tolerance as a model organism for human disease

In recent years, with a global increase in animal welfare awareness and efforts to improve efficiency and reduce the costs of bioresearch, drug discovery, and safety testing, the use of mammalian animals in research has decreased [69]. As alternatives, the use of cultured cells, including induced pluripotent stem cells, predictive analysis using informatics and artificial intelligence, and test systems employing non-mammalian animals, such as fish, insects, and nematodes, has been increasing [70]. Fish are particularly important tools for understanding diseases and developing pharmaceuticals, because they as vertebrates they are more similar to humans than insects or nematodes, and share many organs in common with humans [71–73].

As genome sequencing projects and molecular analyses of teleosts have progressed, various small fish species, including zebrafish [10, 11, 73], medaka [74, 75], goldfish [17], and African turquoise killifish [76] have emerged as valuable model organisms for several research areas, such as toxicology, drug discovery, and human medicine and diseases. Despite the unique biological characteristics of each species, they share commonalities in cellular proliferation, cell cycle, cell division, apoptosis, and fundamental intracellular signaling pathways. These similarities make them excellent model organisms for exploring early development, organ formation, tissue regeneration, infections, and carcinogenesis, and occasionally as models for higher brain functions, such as neurobehavioral and learning capabilities [17, 72, 77, 78]. These small fish models, bolstered by global animal welfare movements, have rapidly gained popularity as alternatives to traditional rodent models such as mice and rats.

However, similar to their rodent counterparts, small fish models have certain limitations. In particular, the requirements of different optimal growth temperatures are often overlooked. Fish are ectothermic, which means that the concept of internally regulated body temperature does not apply to them in its strictest sense. Nevertheless, temperatures higher than normal have been reported to profoundly influence the function of many organs, cause damage to the liver [79–81] and adipose tissues [82] and enhance the immune response [83, 84]. In the field of cancer research, xenograft studies have actively pursued the transplantation of human-derived cancer cells into zebrafish to explore anticancer drugs, therapeutic targets, and mechanisms [85–87]. However, the viability of human-derived cancer cells coexisting with zebrafish in environments of up to 34 °C [88]—and not for extended periods—as true cancer cells, is highly questionable. Numerous reports have highlighted successful examples of human-derived cancer cell transplantation into zebrafish; however, few successes have been noted for deeper cancers, such as pancreatic and prostate cancers. In infectious disease research, small fish species are commonly used as models of human diseases caused by various infectious microorganisms. However, because microorganisms that infect humans generally thrive in environments at 37 °C, there is no strictly fish-based model that accurately replicates human infections [89–91]. Zebrafish are well-known models of tuberculosis. However, when the human pathogen *Mycobacterium tuberculosis* is introduced into zebrafish larvae, the bacterium can only be detected for nine days. As a substitute, another species from the *Mycobacterium genus*, *M. marinum*, has been used as a source of infection [92]. However, *M. marinum* rarely causes respiratory infections in humans, which limits its relevance in modeling human respiratory diseases.

Based on these findings, one of the significant challenges that fish face as model organisms for human diseases is their ability to thrive at high temperatures, such as at 37 °C, which is considered high for most fish species. In addition to *G. rufa*, a few fish, including arowanas and some African cichlids, grow at this temperature. Arowanas, which can reach approximately 1 m in adulthood and live for approximately 10 years, are challenging to manage in laboratory settings due to their size. African cichlids, typically reaching approximately 10 cm in adult size and known for brooding their fertilized eggs inside the mouths of females, are difficult to maintain in mixed-species tanks [93] and have been reported to grow normally only at temperatures up to 35 °C. In contrast, *G. rufa* demonstrates the most robust heat resistance among these species, and is known worldwide for its use as “doctor fish” in spas and hot springs (Table 1). Preliminary tests conducted by our team have shown that it can survive in environments as hot as 40 °C with no observable effect on locomotor activity. *G. rufa* is omnivorous, highly fertile, and reaches adult sizes of 10–15 cm, with juveniles measuring approximately 3 cm, suitable for use in laboratory research. Additionally, the diameter of fertilized eggs is approximately 3 mm, and they are transparent [41], which facilitates microinjections for genetic manipulation and chemical evaluation. Unfortunately, because the genome sequence of *G. rufa* is unknown, reports on its use as a human disease model are lacking. However, similar to zebrafish and medaka, *G. rufa* has the potential to be used as a model organism in medical biology. In addition, the transplantation of human-derived cancer cells, a task that is challenging to achieve in zebrafish, has been successfully demonstrated (data not shown). We believe that *G. rufa* could emerge as a new model organism for human disease research.

**Table 1:** The upper limit temperature to keep various fish

Common name	Scientific name	Upper limit temperature	Size	Breeding
Doctor fish	*Garra rufa*	40 ℃^a^	10–15 cm	Easy
Arowana	*Osteoglossum bcirrhosum*	36 ℃	50–100 cm	Difficult
African cichlids	Cichlidae*^b^	35 ℃	10–15 cm	Difficult (mouth breeding)
Nile Tilapia	*Oreochromis niloticus*	39 ℃	20–60 cm	Difficult (mouth breeding)
Zebrafish	*Danio rerio*	34–35 ℃	3–5 cm	Easy
Medaka	*Oryzias latipes*	34–35 ℃	3–4 cm	Easy

^a^Based on our preliminary data (as mentioned in the text)

^b^Since no studies have yet identified which specific species of African cichlids exhibit high-temperature tolerance, they are referred to at the genus level

### Genome sequencing of *G. rufa*

The limited availability of genomic resources for *G. rufa* hampers genetic manipulations and conduct comprehensive analyses of unique molecular pathways in the species. Recent advances in genome sequencing technology and bioinformatics [94], including long-read sequencing and high-throughput chromosome conformation capture analysis, have made chromosome-level whole-genome sequencing feasible for many organisms [95–98]. To date, 71 cyprinid genome assemblies have been registered in the National Center for Biotechnology Information genome database. Among these genome assemblies, 26 were located at the chromosomal level. The status of genome assemblies of fish belonging to the subfamily Labeoninae, to which *G. rufa* belongs, is detailed in Table 2. Within this subfamily, 11 genome assemblies have been reported, three of which–*Labeo rohita* (GCA_022985175.1), *Cirrhinus molitorella* (GCA_033026305.1), and *Cirrhinus mrigala* (GCA_036247105.1)–are at the chromosome level (Table 2).

**Table 2:** Publicly available genome assemblies in the Labeoninae subfamily

Scientific name	GenBank	Assembly Size (Mb)	Number of chromosome-level scaffolds	Assembly level	Release data
*Cirrhinus cirrhosus *(mrigal carp)	GCA_019207145.1	1,153	-	Contig	Jul, 2021
*Cirrhinus molitorella* (mud carp)	GCA_004028445.1	920	-	Scaffold	Jan, 2019
*Cirrhinus molitorella* (mud carp)	GCA_033026305.1	1,033	25	Chromosome	Oct, 2023
*Cirrhinus mrigala* (mrigala)	GCA_036247105.1	1,057	25	Chromosome	Jan, 2024
*Labeo calbasu* (orange-fin labeo)	GCA_019740295.1	1,042	-	Scaffold	Aug, 2021
*Labeo catla* (catla)	GCA_012976165.1	1,020	-	Scaffold	May, 2020
*Labeo catla* (catla)	GCA_014525385.1	1,232	-	Scaffold	Sep, 2020
*Labeo gonius* (Kuria labeo)	GCA_013461565.1	738	-	Scaffold	Jul, 2020
*Labeo rohita* (rohu)	GCA_004120215.1	1,485	-	Scaffold	Jan, 2019
*Labeo rohita* (rohu)	GCA_017311145.1	1,485	-	Scaffold	Mar, 2021
*Labeo rohita* (rohu)	GCA_022985175.1	1,127	25	Chromosome	Apr, 2022

Cytological studies have determined the chromosome number of *G. rufa* to be 25 [34]. However, direct experimental estimates of the genome size of *G. rufa* are lacking. Given that the genome sizes of *L. rohita* (GenBank accession: GCA_022985175.1) and *C. molitorella* (GenBank accession: GCA_033026305.1), which belong to the same Labeoninae subfamily as *G. rufa*, have been reported to be approximately 1 Gb (Table 2), it is likely that the genome size of *G. rufa* is also approximately 1 Gb.

Transposable elements (TEs) are major components of non-coding regions in animal genomes [99–102]. Although the genome assembly of *G. rufa* has not yet been reported, the TE profiles of other Labeoninae genomes, such as those of *L. rohita*, *C. molitorella*, and *C. mrigala*, provide insights into the likely TE landscape of the *G. rufa* genome. In the *L. rohita* genome, 41.25% has been annotated as repetitive elements, with a notable abundance of LTR retrotransposons and DNA elements [103]. In the *C. molitorella* genome, repetitive elements account for 45.18% of the genome, with DNA elements being the most prevalent (29.37%), followed by LTR retrotransposons (5.55%), long interspersed nuclear elements (LINEs, 4.34%), and short interspersed nuclear elements (SINEs, 0.55%) [104]. Similarly, in the *C. mrigala* genome, repetitive elements account for 48.20% of the genome, with DNA elements constituting 3.67%, and LTR retrotransposons 2.24% [105]. Based on these studies, it is reasonable to hypothesize that the *G. rufa* genome is similarly rich in DNA elements and LTR retrotransposons.

To establish the genomic resources for *G. rufa*, we organized an international consortium that included Mie University, Japan; Badan Riset dan Inovasi Nasional (BRIN), Indonesia; and the University of Vienna, Austria. This consortium focuses on the construction of a chromosome-level assembly of the *G. rufa* genome and characterization of its anatomical and physiological uniqueness [106]. The consortium has established an Agreement on Access and Benefit-sharing for Academic Research, and is endeavoring to establish this locally valuable genetic resource as a novel model organism for biomedical research.

## Conclusion

This review discusses the current scientific and social information on *G. rufa*, highlighting its potential as a new model organism for studying human diseases owing to its ability to thrive at human body temperature, an attribute not observed in other widely used fish models. Since human pharmaceuticals are designed to function at human body temperature and microorganisms that infect humans typically thrive best at 37 °C, *G. rufa*, which also thrives at this temperature, represents a unique advantage as a model for use in biomedical research. Furthermore, the ongoing genome sequencing project for *G. rufa* holds promise for elucidating the mechanisms underlying its high-temperature tolerance, a trait that sets it apart from other Cyprinidae species. These insights could also contribute to fish aquaculture and species conservation in the context of global warming.

## Data Availability

The datasets used and/or analyzed in the current study are available from the corresponding author upon reasonable request.
